# Cellular origin of microRNA‐371a‐3p in healthy males based on systematic urogenital tract tissue evaluation

**DOI:** 10.1111/andr.12595

**Published:** 2019-02-20

**Authors:** W. P. A. Boellaard, A. J. M. Gillis, G. J. L. H. van Leenders, H. Stoop, T. van Agthoven, L. C. J. Dorssers, M. Dinkelman‐Smit, J. L. Boormans, L. H. J. Looijenga

**Affiliations:** ^1^ Department of Urology Erasmus MC Cancer Institute University Medical Center Rotterdam The Netherlands; ^2^ Pathology (LEPO) Erasmus MC Cancer Institute University Medical Center Rotterdam The Netherlands; ^3^ Princess Maxima Center for Pediatric Oncology Utrecht The Netherlands

**Keywords:** microRNA‐371a‐3p, semen biomarker, spermatogenesis, testicular neoplasm, urogenital tract

## Abstract

**Background:**

The microRNA‐371a‐3p (miR‐371a‐3p) has been reported to be an informative liquid biopsy (serum and plasma) molecular biomarker for both diagnosis and follow‐up of patients with a malignant (testicular) germ cell tumor ((T)GCT). It is expressed in all histological cancer elements, with the exception of mature teratoma. However, normal testis, semen, and serum of males with a disrupted testicular integrity without a TGCT may contain miR‐371a‐3p levels above threshold, of which the cellular origin is unknown.

**Objectives:**

Therefore, a series of relevant tissues (frozen and formalin‐fixed paraffin‐embedded (FFPE), when available) from the complete male urogenital tract (i.e., kidney to urethra and testis to urethra) and semen was investigated for miR‐371a‐3p levels using targeted quantitative RT‐PCR (qRT‐PCR).

**Materials and methods:**

In total, semen of males with normospermia (*n* = 11) and oligospermia (*n* = 3) was investigated, as well as 88 samples derived from 32 different patients. The samples represented one set of tissues related to the entire male urogenital tract (11 anatomical locations), three sets for 10 locations, and four sets for six locations.

**Results:**

All testis parenchyma (*n* = 17) cases showed low miR‐371a‐3p levels. Eight out of 14 (57%) semen samples showed detectable miR‐371a‐3p levels, irrespective of the amount of motile spermatozoa, but related to sperm concentration and matched Johnsen score (Spearman's rho correlation coefficient 0.849 and 0.871, *p* = 0.000, respectively). In all other tissues investigated, miR‐371a‐3p could not be detected.

**Discussion:**

This study demonstrates that the miR‐371a‐3p in healthy adult males is solely derived from the germ cell compartment.

**Conclusions:**

The observation is important in the context of applying miR‐371a‐3p as molecular liquid biopsy biomarker for diagnosis and follow‐up of patients with malignant (T)GCT. Moreover, miR‐371a‐3p might be an informative seminal biomarker for testicular germ cell composition.

## Introduction

MicroRNAs (miRNAs) are small, non‐coding single‐stranded RNA molecules about 22 nucleotides long that are involved in post‐transcriptional gene regulation (Lee *et al*., 1993; Reinhart *et al*., 2000; Bentwich *et al*., 2005; Zamore & Haley, 2005). miRNAs are found in diverse organisms, including animals and plants (Ambros, 2003), and are highly stable in various types of human body fluid, including serum, plasma, cerebrospinal fluid, saliva, ejaculate, seminal plasma, and urine (Calin *et al*., 2002; Reis *et al*., 2010).

In 2006, the relevance of a defined set of embryonic stem cell‐associated miRNAs, including miR‐371a‐3p, was identified as potential oncogene for malignant testicular germ cell tumors (TGCT) (Voorhoeve *et al*., 2006). This was subsequently confirmed in a high‐throughput profiling study on TGCTs and unaffected testicular parenchyma, supported by various independent investigations (Gillis *et al*., 2007; Looijenga *et al*., 2007; Palmer *et al*., 2010; Murray *et al*., 2011; Bing *et al*., 2012; Dieckmann *et al*., 2012). Of specific interest is the observation that these miRNAs are also found to be elevated in serum and plasma of patients with malignant (T)GCT compared to healthy individuals, and as such being considered as a promising alternative serum biomarker for diagnosis of (T)GCT in addition to alpha‐fetoprotein (AFP) and human chorionic gonadotropin (hCG) (Gillis *et al*., 2013; Ruf *et al*., 2014; Syring *et al*., 2015; van Agthoven *et al*., 2017; Dieckmann *et al*., 2017; Terbuch *et al*., 2018; Mego *et al*., 2019). This relates both to the initial diagnosis and to the follow‐up of patients with a relapse or non‐responding disease. miR‐371a‐3p is highly expressed in all histological elements of primary as well as metastatic (T)GCT, except for pure teratoma and is absent in other non‐germ cell malignancies (Catto *et al*., 2011; Leao *et al*., 2018). Even in tissue and in serum of patients with the precursor of TGCT (germ cell neoplasia *in situ*, GCNIS), miR‐371a‐3p is reported to be elevated, increasing with the amount of GCNIS cells present (Novotny *et al*., 2012; Radtke *et al*., 2017). Interestingly, the miR‐371a‐3p levels can also be detectable in semen of healthy males, likely related to the same origin as found in normal testicular parenchyma (Gillis *et al*., 2007; Spiekermann *et al*., 2015b). However, the actual source of miR‐371‐3p in healthy males has not yet been defined. Hypothetically, it can be derived from other tissues of the urogenital tract, that is, from kidney to urethra and from testis to urethra as well. The aim of the study was to assess the cellular origin of miR‐371a‐3p in all different anatomical parts of the urogenital tract of males without a TGCT. In addition, a series of semen samples with varying sperm concentration was analyzed.

## Material and Methods

### Ethics statement

The study was approved by the institutional review board Medical Ethics Committee of the Erasmus MC, MEC‐number‐2014‐458. The use of the human samples was in accordance with the “Code for Proper Secondary Use of Human Tissue in The Netherlands,” developed by the Dutch Federation of Medical Scientific Societies (FMWV) (version 2002). The guidelines of the declaration of Helsinki were followed.

### Patient samples

**Figure 1:**
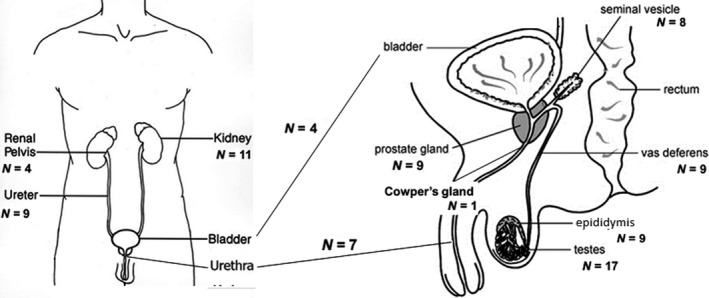
Male urogenital tract from kidney to urethra (left) and from testis to urethra (right). Total number of tissue samples (*n* = 88) of each anatomical part are indicated.

Postoperative tissue samples of 25 different patients and samples from seven autopsies were collected (Fig. 1). Both frozen and formalin‐fixed paraffin‐embedded (FFPE) tissue samples were included. The total cohort consisted of 88 samples: 55 postoperative samples and 33 autopsy samples of the entire urogenital tract. In total, one entire representation of tissues including the male urogenital tract for all the 11 different anatomical locations (kidney, renal pelvis, ureter, bladder, urethra, testis, epididymis, vas deferens, seminal vesicles, prostate, and Cowper's gland), three representations for 10 locations, and four representations for six locations were investigated. The 17 testis samples of patients with non‐malignant disease were scored for spermatogenesis with a Johnsen score (Johnsen, 1970). Semen of 14 cancer‐free subjects attending our clinic for an andrological work‐up was collected by masturbation after three to 5 days of abstinence. All samples were allowed to liquefy at 37 °C for 60 min. before analysis. Semen was analyzed following the World Health Organization (WHO) 2012 criteria. The total motile sperm count (TMSC = volume × concentration × motility) ranging between 0.1 and 261.2, with a mean of 109.8, and a median of 45.1. Thereafter, semen samples were stored at −80 °C. After thawing, semen was immediately processed and analyzed for miR‐371a‐3p levels.

### RNA isolation and investigation

Total RNA from fresh frozen tissue, FFPE samples, and ejaculates (50 μl) was extracted using TRIzol Reagent (Thermo Fisher Scientific, Life Technologies, Bleiswijk, the Netherlands) according to manufacturers’ instruction. Same amount of tissue was used for RNA isolation. Total RNA concentration was measured in triplicate using a NanoDrop ND‐1000 instrument (Isogen Life Science B.V., de Meern, The Netherlands) followed by a quality control (1 ng RNA input) using a qRT‐PCR with TaqMan assays for RNU48 (001006) and miR‐20a‐5p (000580). RNA samples of suitable quality (Cq < 28) were subjected to miRNA profiling. Total RNA (10 ng input) was converted into cDNA using a TaqMan miRNA RT‐Kit and TaqMan miRNA RT‐primers for miR‐371a‐3p (002124), and the normalizers miR‐20a‐5p (semen), and RNU48 (tissue). After cDNA synthesis, efficiency was checked. miRNA levels were determined on a TaqMan 7500HT Real‐Time PCR machine and are depicted as 40 (the highest cycle) minus Ct observed. The 40‐Ct, scale log_2_ representation was used because heterologous tissue samples were compared. The 2^−∆∆CT^ approach is specifically useful for comparison of individual samples to a selected control, not applicable for this study. All devices and kits are purchased from Thermo Fisher Scientific. miRNA levels in tissues were normalized using the average levels of all samples of RNU48, and miR‐levels in semen were normalized using the average of miR‐20a‐5p.

### Software and statistics

Microsoft Excel 2010 and IBM spss statistics V21.0 were used for analysis. miRNA levels in tissues were normalized using the average levels of all samples of RNU48, and miR‐levels in semen were normalized using the average of miR‐20a‐5p.

## Results

**Figure 2:**
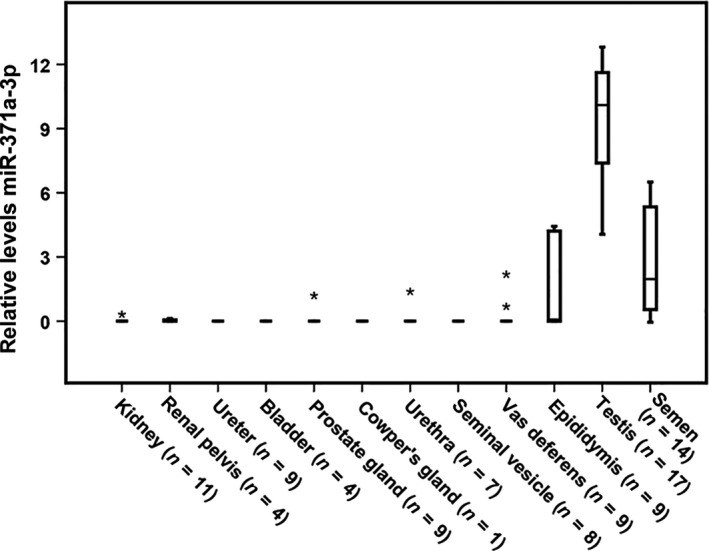
Detection of miR‐371a‐3p in the male urogenital tract. Boxplots of the relative levels of miR‐371a‐3p are presented (40‐Ct, scale log2), normalized with RNU48. Kidney (*n* = 11), renal pelvis (*n* = 4), ureter (*n* = 9), bladder (*n* = 4), prostate gland (*n* = 9), Cowper's gland (*n* = 1), urethra (*n* = 7), seminal vesicle (*n* = 8), vas deferens (*n* = 9), epididymis (*n* = 9), testis (*n* = 17), semen (*n* = 14), normalized with miR‐20a‐5p. The box marks the first and third quartiles. Horizontal lines mark median values; outliers are indicated with an asterisk. The whiskers indicate the minimum and maximum values.

**Figure 3:**
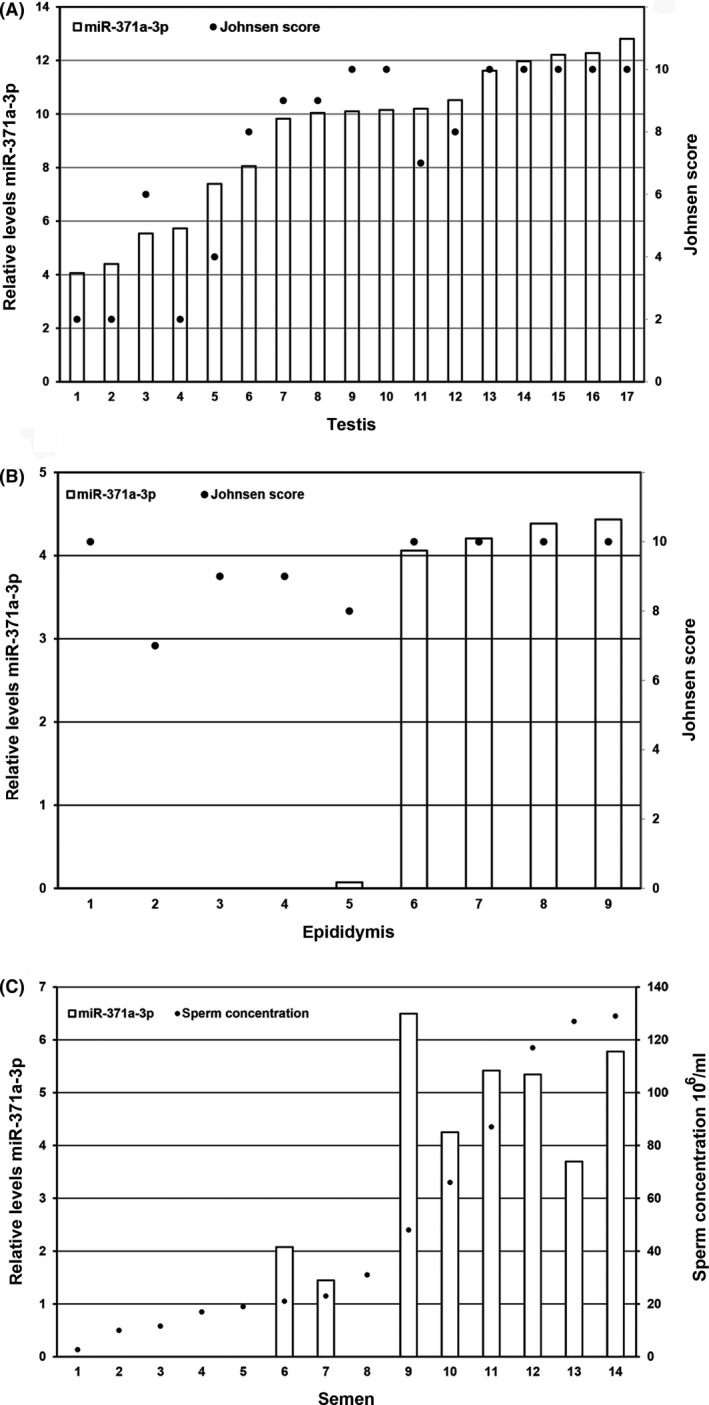
(A) Detection of miR‐371a‐3p in testis in relation to Johnsen score. Left *Y*‐axis 40‐Ct, scale log2, right *y*‐axis Johnsen score (Spearman's rho correlation coefficient (0.871, *p* = 0.000). (B) Detection of miR‐371a‐3p in epididymis in relation to Johnson score. Left *Y*‐axis 40‐Ct, scale log2, right *y*‐axis Johnsen score. (C) Detection of miR‐371a‐3p in semen. The measurements in the 14 semen samples normalized with miR‐20a‐5p (samples 1–3 have oligospermia with sperm concentrations below 12 million/ml; numbers 4–8 have a sperm concentration between 17 million/ml and 35 million/ml; and numbers 9–14 have a sperm concentration between 48 and 129 million/ml). Left *Y*‐axis 40‐Ct, scale log2, right *y*‐axis sperm concentration, linear scale. The miR‐371a‐3p level increased with the sperm concentration (Spearman's rho correlation coefficient 0.849, *p* = 0.000).

In total, 88 tissue samples were analyzed (Figs 1 and 2). Testis parenchyma (*n* = 17) all showed low levels of miR‐371a‐3p. The miR‐371a‐3p levels increased significantly with the Johnsen score (Spearman's rho correlation coefficient 0.871, *p* = 0.000) (Fig. 3A). Epididymis tissue samples (five out of nine) showed the presence of miR‐371a‐3p but lower levels than observed in testicular tissue. Four epididymis samples did not show miR‐371a‐3p even though the Johnsen score was above eight (Fig. 3B). All other tissues lacked miR‐371a‐3p levels detectable above threshold (Fig. 2). No differences were found between 64 frozen and 24 FFPE tissues (Table S1). The 14 semen samples were normospermia in 11 and oligospermia in three. Concentrations ranged between 2.7 and 129 million sperm cells per ml with a mean of 50.7 (Fig. 3C). Eight out of 14 (57%) semen samples showed miR‐371a‐3p, irrespective of the mean amount of motile sperm cells, but with a minimum concentration of 21 million sperm cells per ml (Fig. 3C, sample 6, 7, 9–14). The miR‐371a‐3p level increased with the sperm concentration (Spearman's rho correlation coefficient 0.849, *p* = 0.000).

## Discussion and Conclusion

This study indicates that in healthy males, the germ cell compartment is the cellular origin of miR‐371a‐3p. In addition, miR‐371a‐3p seemed to correlate with the sperm concentration, the output of spermatogenesis and therefore a proxy for the germ cell composition. Individual miRNAs from the cluster miR‐371‐373 on chromosomal location 19q13 are expressed in all malignant (T)GCTs, regardless of patient age, tumor site, and subtype (Murray *et al*., 2015, 2016b). The cluster miR‐371‐373 is expressed in all histological elements of primary as well as metastatic TGCT, except for pure teratoma (Voorhoeve *et al*., 2006; Cheng *et al*., 2018; Leao *et al*., 2018; Terbuch *et al*., 2018). The different tumor subtypes display differential expression of miR‐371a‐3p, depending on the level of differentiation (Vilela‐Salgueiro *et al*., 2018). This is in line with our earlier studies (Gillis *et al*., 2013). Tumor load seems to play a role in the level of serum miR‐371a‐3p. miR‐371a‐3p levels in serum are increasing with the amount of GCNIS cells in the pre‐invasive stage of TGCT and also with primary tumor size in localized disease (Dieckmann *et al*., 2012; Novotny *et al*., 2012; Radtke *et al*., 2017). This relation is confirmed by the reduction of miR‐371a‐3p levels after tumor load is decreased by orchiectomy in GCNIS and localized disease (Gillis *et al*., 2013; Syring *et al*., 2015; Radtke *et al*., 2017, 2018). Moreover, miR‐371a‐3p levels increase with dissemination degree in metastasized disease and levels decrease after chemotherapy response (Dieckmann *et al*., 2012, 2017). In addition, proximity to the tumor seems related to the miR‐371a‐3p levels as testicular vein blood showed higher levels than cubital vein blood (Spiekermann *et al*., 2015a). Even cerebrospinal fluid, pleural effusion, and hydrocele fluid next to tumor contain high levels miR‐371a‐3p (Dieckmann *et al*., 2016; Murray *et al*., 2016a). In semen of healthy males, miR‐371a‐3p is detectable, likely related to the origin as found in normal testicular parenchyma (Gillis *et al*., 2007; Spiekermann *et al*., 2015a). Theoretically, it can be derived from other tissues of the urogenital tract, that is, from kidney to urethra and from testis to urethra as well. To elucidate the source of miR‐371a‐3p in the ejaculate of healthy men, we analyzed the entire urogenital tract for miR‐371a‐3p levels by qRT‐PCR. This is because compounds in the ejaculate can be deposited by these organs draining on the urogenital tract. In our series, no miR‐371a‐3p was found in tissue derived from the kidney, renal pelvis, ureter, bladder, urethra, vas deferens, seminal vesicles, prostate, or Cowper's gland, whereas both in the testis and in the epididymis, miR‐371a‐3p levels were found, suggesting that the gonadal germ cell compartment is the source of origin. This was supported by the finding of a positive correlation between miR‐371a‐3p, sperm concentration, and the Johnsen score. Both increased sperm concentration and Johnsen score indicate higher levels of gonadal cells. We speculate that the low levels of miR‐371a‐3p detected in the epididymis in patients with a normal testicular function might have been caused by epididymal obstruction.

A recent publication on seminal miR‐371a‐3p in TGCT patients showed seminal plasma levels of stage I TGCT patients to have an opposite trend to serum levels. Preoperatively stage I TGCT patients had lower seminal miR‐371a‐3p levels than healthy controls and seminal plasma levels normalized after orchiectomy to levels comparable to healthy controls (Pelloni *et al*., 2017). Possibly, miR‐371a‐3p levels are influenced by testicular integrity like we found in our previous studies on males with a non‐malignant testicular tumor (van Agthoven & Looijenga, 2017). Our results on semen of healthy males are an important start for further exploration of the role of seminal miR‐371a‐3p levels in healthy and diseased males. Moreover, a relation between the amount of germ cells and miR‐371a‐3p levels was found. Even in patients histologically classified as Sertoli cell‐only syndrome (i.e., Johnsen score 2), miR‐371a‐3p was found. Possibly, these patients had an incomplete Sertoli cell‐only pattern with focal spermatogenesis. Thus, miR‐371a‐3p might be informative as a liquid biopsy of spermatogenic function of the testis as well, discriminating patients who will have a chance of surgical sperm retrieval on testicular sperm extraction (TESE) (Vernaeve *et al*., 2006; Li *et al*., 2012).

Our study demonstrates for the first time that the miR‐371a‐3p in normal adult males is solely derived from the germ cell compartment. This finding can be used in further investigations in the role of miR‐371a‐3p as a liquid biopsy for GCNIS detection and follow‐up of TGCT. A relation between spermatogenesis and miR‐371a‐3p was found. Further research is needed to define the role of seminal miR‐371a‐3p in predicting a successful TESE.

## Authors’ Contributions

WPAB and LHJL conceived and designed the experiments; AJMG, HS, and GJLHvL performed the experiments; TvA and LCJD analyzed the data; WPAB, MDS, JLB, TvA, and LHJL contributed to the writing of the manuscript.

## Supporting information


**Table S1** Raw results of the qRT‐PCR analysis of the tissue samples included (both FFPE and frozen).
